# Antibiotic Consumption and Healthcare-Associated Infection Surveillance in a Multi-Unit Emergency Hospital in Romania: A Retrospective Observational Study

**DOI:** 10.3390/medicina62061171

**Published:** 2026-06-16

**Authors:** Mioara-Calipsoana Matei, Valeriu-Aurelian Chirica, Marcel Ifrim, Cristina Morariu, Doina Spaiuc, Alina Manole, Mihaela Moscalu

**Affiliations:** 1 Grigore T. Popa University of Medicine and Pharmacy Iasi, 700115 Iasi, Romania; mat_calipso@yahoo.com (M.-C.M.); mm_alina@yahoo.com (A.M.); moscalu.mihaela@gmail.com (M.M.); 2 “Dr. Iacob Czihac” Military Emergency Clinical Hospital, 700483 Iasi, Romania; ifrim.marcel@yahoo.com (M.I.); morariu.cristina@yahoo.com (C.M.); doina.spaiuc@yahoo.com (D.S.); 3 Iasi Regional Public Health Center, 700465 Iasi, Romania

**Keywords:** antibiotic consumption, antimicrobial stewardship, AWaRe classification, healthcare-associated infections, infection control, intensive care unit, hospital epidemiology, surveillance, Romania

## Abstract

*Background and Objectives*: Healthcare-associated infections (HAIs) remain a major challenge in emergency hospital settings, where high patient turnover and empirical antibiotic use may contribute to the emergence and spread of multidrug-resistant organisms. Monitoring antibiotic consumption is essential for antimicrobial stewardship and infection prevention. This study evaluated antibiotic consumption patterns across multiple hospital units and explored their ecological relationship with HAI rates. *Materials and Methods*: A retrospective observational study was conducted in a tertiary-level emergency hospital in Romania between 1 January 2021 and 31 October 2025. Antibiotic consumption was quantified using Defined Daily Dose per 100 bed-days (DDD/100 bed-days) according to World Health Organization (WHO) methodology and categorized using the WHO Access, Watch, and Reserve (AWaRe) classification. HAI data were collected using standardized surveillance definitions. Statistical analyses were primarily descriptive and exploratory and included graphical trend assessment, simple linear regression for temporal trend description, and Spearman correlation analysis for exploratory ecological co-variation assessment. *Results*: Antibiotic consumption showed substantial variability across hospital units, without a consistent temporal trend over the study period. The Watch group predominated over the Access group from 2023 onward, while Access antibiotics remained below the WHO-recommended 60% threshold. The highest antibiotic consumption was observed in the Medical Wards, followed by Surgical Wards and the Intensive Care Unit. A total of 27 HAIs were identified (0.27 per 1000 patient-days), with the highest incidence observed in the ICU. The most frequent infections were *Clostridioides difficile* infections (33.3%) and catheter-associated urinary tract infections (29.6%). Exploratory ecological analyses did not identify robust associations between total antibiotic consumption and HAI rates across hospital units. A numerically elevated co-variation was observed between fluoroquinolone consumption and *Clostridioides difficile* infection incidence; however, this finding should be interpreted strictly as exploratory and hypothesis-generating. *Conclusions*: Antibiotic use varied across hospital units, with predominance of broad-spectrum agents and suboptimal adherence to WHO AWaRe targets. Reported HAI incidence remained low and should be interpreted within the limitations of routine surveillance systems and potential under-ascertainment. These findings support the value of continuous institutional surveillance of antibiotic use and HAIs while highlighting the limitations of aggregated ecological analyses.

## 1. Introduction

Healthcare-associated infections (HAIs) remain a major challenge for modern healthcare systems, contributing to prolonged hospitalization, increased morbidity and mortality, and substantial economic burden. Recent European and global surveillance reports indicate that HAIs continue to affect a substantial proportion of hospitalized patients, particularly in acute-care settings, while antimicrobial resistance (AMR) further complicates prevention and treatment strategies [1,2]. Emergency and acute-care hospitals are especially vulnerable because of high patient turnover, clinical complexity, frequent use of invasive devices, and the need for rapid empirical antibiotic therapy [1,3]. In the present study, the term “emergency hospital” refers to a tertiary-level acute-care hospital with integrated emergency admission pathways and multiple clinical specialties, rather than solely an emergency department. These factors facilitate the emergence and transmission of both HAIs and multidrug-resistant organisms (MDROs) [1,4].

Antibiotic consumption is a major driver of AMR, and inappropriate or excessive use of broad-spectrum agents is associated with the emergence and selection of resistant pathogens [5,6]. Global studies have reported increasing trends in antibiotic consumption, with important regional variations and projections indicating continued growth [7,8]. At the hospital level, antimicrobial use has been associated with resistance patterns and HAI epidemiology, particularly in high-risk settings such as intensive care units and medical and surgical wards, where prescribing practices may influence antimicrobial selection pressure [6,9,10,11,12,13,14,15,16,17,18,19].

In this context, antimicrobial stewardship (AMS) programs play a central role in optimizing antibiotic use, promoting evidence-based therapy, and aligning prescribing practices with local epidemiology [20,21]. A core component of AMS is routine surveillance of antibiotic consumption, which provides data to guide targeted interventions and support infection prevention and control (IPC) activities [22,23,24]. International frameworks recommend standardized indicators, including Defined Daily Dose (DDD) metrics and the World Health Organization (WHO) Access, Watch, and Reserve (AWaRe) classification, to improve comparability across healthcare settings [25,26,27,28,29].

Despite these recommendations, many hospitals, particularly multi-unit emergency and acute-care facilities, lack integrated systems for consistent monitoring and comparison of antibiotic consumption across clinical units [3,30]. The complexity of care in these settings may favor prolonged empirical prescribing without systematic reassessment, thereby increasing unnecessary antibiotic exposure and selective pressure for resistant organisms [7,9]. Strengthening surveillance of antimicrobial use may therefore support improved prescribing practices and infection prevention efforts [6,22].

Previous studies have shown that analysis of antibiotic consumption can help identify high-risk units, describe prescribing patterns, and explore ecological associations with HAI incidence [10,20,30]. However, evidence from multi-unit emergency hospital settings remains limited, and relatively few studies have evaluated antibiotic-use surveillance within integrated AMS and IPC activities in these environments [3,23,31,32]. Recent surveillance reports continue to show high levels of antimicrobial use in acute-care hospitals, frequently exceeding recommended targets [1,33]. International organizations increasingly emphasize standardized approaches for monitoring antibiotic consumption as part of coordinated AMS and IPC strategies in hospital settings [25,26,34,35].

The aim of this study was to characterize antibiotic consumption patterns across multiple hospital units and to explore their ecological association with HAI incidence in a tertiary emergency hospital setting within an exploratory observational framework. This study was not designed to establish causal relationships, but rather to provide hospital surveillance data on antimicrobial use and infection dynamics during routine clinical practice. By integrating antimicrobial consumption and HAI surveillance data, this study contributes to AMS and IPC activities and provides a framework for unit-level monitoring in acute-care hospital settings.

The aim of this study was to characterize antibiotic consumption patterns across multiple hospital units and to explore their ecological relationship with HAI incidence in a tertiary emergency hospital setting within an exploratory observational framework. The study was not designed to establish causal relationships, but rather to provide institutional surveillance data regarding antimicrobial use and HAI patterns during routine clinical practice. By integrating antimicrobial consumption and HAI surveillance data, this study contributes to the understanding of unit-level surveillance patterns relevant to AMS and IPC activities in acute-care hospital settings.

## 2. Materials and Methods

### 2.1. Setting

The study was conducted at the “Dr. Iacob Czihac” Military Emergency Clinical Hospital, a tertiary-level acute-care hospital in Romania providing continuous emergency admission and multidisciplinary inpatient services for both civilian and military populations.

The hospital includes high-risk units such as the Intensive Care Unit (ICU), Surgical Wards (including a small number of gynecology beds), Medical Wards, Emergency Department, and a Day-care Hospitalization Unit, with approximately 200 inpatient beds. The institution does not include a pediatric unit; therefore, no pediatric patients were included.

Infection prevention and control (IPC) activities are coordinated by a multidisciplinary team including an epidemiologist, infectious diseases specialist, microbiologist, pharmacist, and infection-control nursing staff. HAI surveillance is performed according to national guidelines using standardized European Centre for Disease Prevention and Control (ECDC) definitions applied consistently across all hospital units.

Antimicrobial stewardship (AMS) activities are part of routine hospital practice and include formulary restriction policies, infectious diseases consultation, audit-and-feedback processes, and periodically updated prescribing guidelines. These activities were not implemented or evaluated as a structured intervention within the present study.

### 2.2. Study Design

This retrospective observational study evaluated antibiotic consumption patterns and their ecological relationships with healthcare-associated infection (HAI) rates across hospital units. The study period extended from 1 January 2021 to 31 October 2025, allowing assessment of temporal trends.

The study was conducted in accordance with the Strengthening the Reporting of Observational Studies in Epidemiology (STROBE) guidelines. No experimental interventions were introduced by the research team.

All data were derived from routinely collected institutional sources, including electronic medical records, HAI surveillance reports, microbiology laboratory databases, and electronic pharmacy records. Data extraction and analysis were performed retrospectively from anonymized and aggregated datasets obtained as part of routine clinical and surveillance activities.

The study adhered to ethical standards for research involving anonymized and aggregated patient data and was approved by the institutional ethics committee (approval no. 73/E3333, 11 July 2025). Given the use of anonymized routine surveillance data, informed consent was waived.

### 2.3. Data Collection

#### 2.3.1. Descriptive Data

Descriptive patient-level data were collected to characterize the study population and contextualize healthcare-associated infection (HAI) patterns. For patients with HAIs, the following variables were recorded: sex, age, hospital unit at the time of infection diagnosis, and time from hospital admission to microbiological diagnosis.

Clinical variables included the type of HAI and the type of clinical specimen (e.g., urine, respiratory tract, surgical site, sputum, and stool samples). Microbiological data were extracted from the laboratory information system and included pathogen identification and antimicrobial susceptibility testing (AST) results.

Microbiological identification was performed using standard culture-based methods, Gram staining, and biochemical testing (API systems when required). MALDI-TOF mass spectrometry and fully automated identification systems were not routinely available during the study period.

Antimicrobial susceptibility testing was performed using the disk diffusion (Kirby–Bauer) method and interpreted according to Clinical and Laboratory Standards Institute (CLSI) guidelines (2021–2025). Multidrug-resistant organisms (MDROs) were defined according to the consensus criteria proposed by Magiorakos et al. (2012) and national surveillance guidance [36,37]. Only routinely tested antimicrobial categories were considered for MDRO classification. Intrinsic resistance patterns and antimicrobial categories not routinely tested for a given microorganism were excluded. Antimicrobial categories were included only when at least one representative agent had been tested for the respective isolate.

All data were anonymized and analyzed in aggregated form.

#### 2.3.2. Antibiotic Consumption Data

Antibiotic utilization data were extracted from the hospital pharmacy electronic dispensing system, which records all dispensed systemic antibacterial agents by hospital unit and date. Accordingly, antibiotic consumption reflects dispensed rather than directly administered antibiotics at the patient level. Dispensing records were used as the primary surveillance source due to their consistent availability across all hospital units throughout the study period.

Antibiotics were classified according to the World Health Organization (WHO) Anatomical Therapeutic Chemical (ATC) classification system (J01). Consumption was quantified using Defined Daily Doses (DDD) and expressed as DDD per 100 bed-days (DDD/100 bed-days), in accordance with WHO ATC/DDD methodology. This metric was used because it represents the standardized approach recommended for hospital antimicrobial surveillance and facilitates comparability with international surveillance systems and published studies.

When available, information regarding mean duration of therapy per treated patient was extracted from prescription records. However, patient-level Days of Therapy (DOT) data were not consistently available across all hospital units and therefore were not included as a standardized metric in the present analysis.

Antibiotics were additionally grouped into predefined therapeutic classes, including penicillins, cephalosporins, carbapenems, fluoroquinolones, aminoglycosides, glycopeptides, polymyxins, oxazolidinones, tetracyclines, nitrofurans, and other antimicrobial categories detailed in Appendix Table A4. These groupings were used exclusively for antibiotic consumption analyses and were not applied to MDRO classification or AST interpretation. Consumption trends were analyzed at the hospital-unit level.

Antibiotic consumption was further categorized according to the World Health Organization (WHO) Access, Watch, and Reserve (AWaRe) classification framework. The AWaRe system groups antibiotics into Access (first-line agents with lower resistance potential), Watch (agents with higher resistance selection potential), and Reserve (last-resort antibiotics for multidrug-resistant infections) categories. The framework supports antimicrobial stewardship and surveillance, with WHO recommending that at least 60% of total antibiotic consumption should originate from the Access group [25,26].

AWaRe categorization was applied at the individual antibiotic molecule level. The WHO AWaRe 2023 classification was used for the 2021–2024 period, while the WHO AWaRe 2025 classification was applied for 2025 data. No harmonization across classification versions was performed.

#### 2.3.3. Healthcare-Associated Infection Data

In this study, the primary outcome variable was healthcare-associated infection (HAI). HAIs were defined as infections acquired during healthcare delivery that were not present or incubating at the time of admission and that fulfilled the European Centre for Disease Prevention and Control (ECDC) surveillance criteria applicable to the respective infection type [38]. Outcome measures included the annual number of HAIs and HAI incidence rates expressed as cases per 1000 patient-days. HAI data were retrieved from the hospital surveillance system using standardized ECDC definitions [38] applied across all hospital units during the study period (2021–2025). Case identification relied on specific clinical criteria, timing relative to admission, and invasive device exposure. Additionally, microbiological data—including pathogen identification and the detection of multidrug-resistant organisms (MDROs)—were extracted from the surveillance database.

HAIs were classified into the following categories: *Clostridioides difficile* infection (CDI), catheter-associated urinary tract infection (CAUTI), ventilator-associated pneumonia (VAP), surgical site infection (SSI), bloodstream infection (BSI), and other respiratory infections. The category “other respiratory infections” also included hospital-acquired SARS-CoV-2 infections identified according to institutional and national surveillance criteria.

CAUTI was defined according to ECDC criteria as urinary tract infection occurring in patients with an indwelling urinary catheter in place for more than 48 h at the time of infection onset or within the ECDC-defined risk window following catheter removal. Community-acquired urinary tract infections were excluded from this category. All healthcare-associated urinary tract infections identified during the study period occurred in patients with indwelling urinary catheters and were classified as CAUTI within the institutional surveillance framework.

HAI data included the number of infections per unit and per year, distribution of infection types, and incidence rates expressed as cases per 1000 patient-days. For device- and procedure-associated infections (including VAP, CAUTI, and SSI), incidence rates were additionally calculated using procedure- and device-based denominators when available, although these were not consistently recorded across all hospital units.

CDI was analyzed separately from device-associated infections in accordance with ECDC surveillance methodology. Laboratory diagnosis of CDI was performed using standardized institutional laboratory protocols based on toxin detection.

#### 2.3.4. Additional Variables

To contextualize observed antibiotic consumption and HAI trends, additional institutional variables were collected, including hospital bed occupancy rates and antimicrobial stewardship (AMS) activities implemented during the study period.

Between 2021 and 2025, AMS activities were progressively expanded across the institution, particularly in high-risk areas such as the Intensive Care Unit (ICU) and Surgical Wards. These activities included formulary restriction policies, infectious diseases specialist consultation, and audit-and-feedback processes targeting selected broad-spectrum antibiotics in the ICU and Surgical Wards. Institutional antibiotic prescribing recommendations based on local microbiological surveillance data were implemented at the end of 2022 and updated in 2024. Educational initiatives regarding the WHO AWaRe classification and core antimicrobial stewardship principles were progressively expanded after 2022. These developments reflect the progressive evolution of routine antimicrobial stewardship practices over time and are presented for contextual purposes only, without implying a direct causal relationship with antibiotic consumption trends.

These activities represented routine institutional antimicrobial stewardship and infection prevention practices and were not implemented, standardized, or evaluated as part of a controlled interventional study. Consequently, the present study was not designed to assess causal or independent effects of specific AMS measures on antibiotic consumption or HAI trends.

### 2.4. Study Population

The study included all patients admitted during the observation period (1 January 2021–31 October 2025) who completed a hospital episode of care, defined as discharge home, transfer to another healthcare facility or ward, or in-hospital death. Overall, 24,075 patients met these inclusion criteria, with the following annual distribution: 3456 in 2021, 4624 in 2022, 5472 in 2023, 5679 in 2024, and 4844 in 2025 (January–October).

Patient admissions were distributed across clinical units, with the highest numbers recorded in Medical Wards, Surgical Wards, and ICU. Medical Wards included Internal Medicine, Dermatology, Neurology, and Psychiatry, while Surgical Wards comprised General Surgery, Orthopedics, Ophthalmology, and Ear, Nose, and Throat (ENT). Additional data were obtained from the Day-care Hospitalization Unit and the Emergency Department.

During the study period, antibiotic therapy was dispensed to 10,496 hospitalized patients, representing a subset of the total study population: 1831 in 2021, 2048 in 2022, 2225 in 2023, 2556 in 2024, and 1836 in 2025 (January–October).

Lower numbers of antibiotic-treated patients were observed in the Day-care Hospitalization Unit (*n* = 28) and the Emergency Department (*n* = 22), reflecting the shorter duration and lower complexity of care episodes in these settings.

As all eligible hospital admissions during the study period were included, no formal sample size calculation was performed, consistent with the retrospective observational design of the study.

### 2.5. Statistical Analysis

Statistical analysis was primarily descriptive and exploratory. Continuous variables were summarized using means and standard deviations (SD) or medians and interquartile ranges (IQR), as appropriate. Categorical variables were presented as frequencies and percentages.

Antibiotic consumption was expressed as Defined Daily Dose per 100 bed-days (DDD/100 bed-days), according to the WHO Anatomical Therapeutic Chemical (ATC)/DDD methodology, to ensure comparability with international antimicrobial consumption surveillance systems.

Temporal patterns in antibiotic consumption (2021–2025) were explored descriptively, with year-to-year variations illustrated graphically. Where appropriate, simple linear regression was used solely as an exploratory tool to summarize the overall direction of temporal change, reporting regression coefficients (β) and coefficients of determination (R^2^), without inferential interpretation.

The distribution of antibiotic consumption according to the WHO Access, Watch, and Reserve (AWaRe) classification was analyzed descriptively over time.

HAI rates were calculated as cases per 1000 patient-days. For device- and procedure-associated infections, incidence rates were additionally calculated using procedure- and device-based denominators when available.

Given the ecological design of the study and the limited number of annual aggregated observations (2021–2025), associations between antibiotic consumption and HAI rates were assessed using Spearman nonparametric correlation analysis at both hospital and unit levels as an exploratory descriptive measure.

All analyses were conducted within a descriptive and exploratory framework. No causal or individual-level inferences were made due to the aggregated nature of the data and the limited number of time points.

## 3. Results

### 3.1. Characteristics of Hospital Units

The study included multiple clinical units with varying patient profiles and levels of care complexity. The hospital had an overall capacity of approximately 200 beds, with the largest units being the Surgical Wards (88 beds) and the Medical Wards (87 beds). The Intensive Care Unit (ICU) comprised 15 beds and managed critically ill patients, while smaller units included the Emergency Department and the Day-care Hospitalization Unit.

Average occupancy rates ranged from 33.3% in the ICU to 43.2% in the Medical Wards, while the Day-care Hospitalization Unit operated at full turnover capacity. Healthcare-associated infection (HAI) surveillance was conducted across all hospital units based on institutional protocols aligned with ECDC definitions. Given the higher-risk patient populations and greater use of invasive devices, enhanced surveillance and focused monitoring of device- and procedures-associated infections and multidrug-resistant organisms (MDROs) were implemented in the ICU and Surgical Wards (Table 1).

### 3.2. Antibiotic Consumption Trends

Antibiotic consumption showed marked inter-annual variability during the study period (2021–2025). Total antibiotic consumption reached a pronounced peak in 2022 (1605.11 DDD/100 bed-days), followed by lower values in 2023 and 2024, with partial data available for 2025 (January–October: 464.31 DDD/100 bed-days) (Table 2).

Given the short and non-monotonic time series, temporal patterns were explored using simple linear regression as a descriptive approach to summarize the overall direction of change. Considering the limited number of annual observations, this analysis was not intended for inferential purposes. The results indicated an apparent slight decreasing pattern in total antibiotic consumption (β = −100.535), although model fit was low (R^2^ = 0.081), reflecting substantial inter-annual variability and a non-linear temporal trend (Table 3).

**Figure 1:**
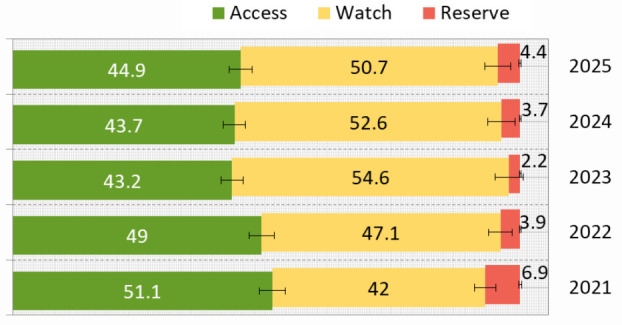
Share of total antibiotic consumption according to the WHO AWaRe classification (2021–2025). The figure illustrates the annual proportional distribution of Access, Watch, and Reserve antibiotics in total hospital antibiotic consumption. Error bars represent 95% confidence intervals. Data for 2025 represent the period January–October and are not directly comparable with full-year estimates.

The distribution of antibiotic consumption according to the WHO AWaRe classification varied over time (Figure 1). The proportion of Access antibiotics decreased from 51.1% in 2021 to 43.2% in 2023, with a value of 44.9% observed in 2025. In contrast, Watch antibiotics increased from 42.0% in 2021 to 54.6% in 2023, remaining at 50.7% in 2025. Reserve antibiotics accounted for a consistently small proportion of total consumption throughout the study period (2.2–6.9%). Overall, from 2023 onwards, Watch antibiotics exceeded Access antibiotics in relative proportion.

**Figure 2:**
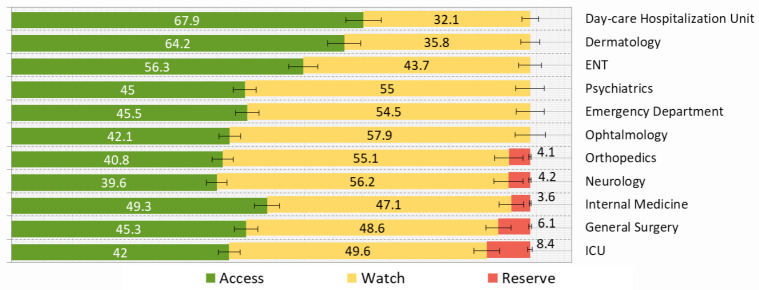
Distribution of antibiotic consumption according to the WHO AWaRe classification across hospital units (%). The figure illustrates the proportional distribution of Access, Watch, and Reserve antibiotics across hospital units during the study period. AWaRe categorization was performed at the individual antibiotic molecule level using the WHO AWaRe 2023 classification for the 2021–2024 period and the WHO AWaRe 2025 classification for 2025. Error bars represent 95% confidence intervals.

AWaRe distribution also varied across hospital units (Figure 2). Higher proportions of Access antibiotics were observed in the Day-care Hospitalization Unit (67.9%), Dermatology (64.2%), and ENT (56.3%). In contrast, higher proportions of Watch antibiotics were observed in higher-acuity clinical units, including the ICU (49.6%), General Surgery (48.6%), and Internal Medicine (47.1%). Reserve antibiotics represented a consistently small fraction of total use across all units, with the highest proportions observed in the ICU (8.4%) and General Surgery (6.1%), followed by Neurology (4.2%), Orthopedics (4.1%), and Internal Medicine (3.6%).

### 3.3. Antibiotic Consumption by Hospital Unit

Antibiotic consumption varied substantially across hospital units. The Medical Wards accounted for the largest proportion of total antibiotic use (61.58%), followed by the Surgical Wards (22.48%) and the Intensive Care Unit (ICU) (12.08%). The Emergency Department (1.41%) and Day-care Hospitalization Unit (2.43%) contributed minimally to overall consumption (Table 4).

At the hospital level, the most frequently used antibiotic classes were fluoroquinolones (561.42 DDD/100 bed-days), penicillins (365.17 DDD/100 bed-days), extended-spectrum cephalosporins (327.71 DDD/100 bed-days), and penicillins combined with β-lactamase inhibitors (246.38 DDD/100 bed-days), which together accounted for the majority of total consumption (Table 4).

Distinct prescribing patterns were observed across hospital units (Table 5). In the Medical Wards, the highest consumption was observed for fluoroquinolones (370.47 DDD/100 bed-days), followed by penicillins (204.38 DDD/100 bed-days) and β-lactamase inhibitor combinations (166.68 DDD/100 bed-days). These wards also contributed the largest absolute consumption across most antibiotic classes.

The Surgical Wards showed intermediate consumption levels, with fluoroquinolones (116.18 DDD/100 bed-days), penicillins (92.86 DDD/100 bed-days), and extended-spectrum cephalosporins (76.16 DDD/100 bed-days) representing the predominant classes.

The ICU exhibited a lower absolute share of total hospital antibiotic consumption but a higher relative use of broad-spectrum and Reserve antibiotics. In this unit, oxazolidinones (42.56 DDD/100 bed-days), carbapenems (34.21 DDD/100 bed-days), glycopeptides (33.38 DDD/100 bed-days), and polymyxins (2.10 DDD/100 bed-days) were more prominent than in other hospital units. Fluoroquinolones (58.91 DDD/100 bed-days) and extended-spectrum cephalosporins (33.85 DDD/100 bed-days) were also frequently used.

The Emergency Department and Day-care Hospitalization Unit showed substantially lower antibiotic consumption and a narrower antimicrobial spectrum, with fluoroquinolones, penicillins, and extended-spectrum cephalosporins representing the main classes in both settings.

**Figure 3:**
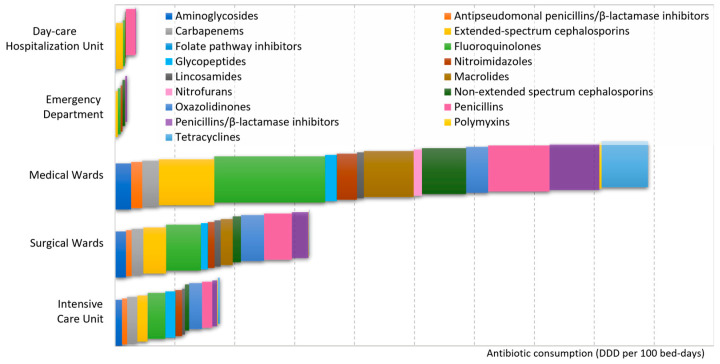
Distribution of antibiotic consumption by class and hospital unit expressed as DDD per 100 bed-days. Internal Medicine demonstrated the highest overall antibiotic consumption, whereas the ICU showed greater relative use of broad-spectrum and Reserve antibiotics, including carbapenems, glycopeptides, polymyxins, and oxazolidinones. The Emergency Department and Day-care Hospitalization Unit exhibited lower overall antibiotic consumption and a narrower spectrum of antibiotic use.

Overall, antibiotic consumption showed marked heterogeneity across hospital units, ranging from higher absolute use in Medical Wards to relatively increased use of broad-spectrum and Reserve antibiotics in higher-acuity units managing critically ill and postoperative patients, particularly the ICU and Surgical Wards (Table 5; Figure 3).

### 3.4. Healthcare-Associated Infections (HAIs)

A total of 27 HAIs were recorded during the study period, corresponding to an overall rate of 0.27 cases per 1000 patient-days. The highest HAI rate was observed in the ICU (1.13 per 1000 patient-days), followed by the Surgical Wards (0.26 per 1000 patient-days) and the Medical Wards (0.05 per 1000 patient-days). In absolute numbers, most HAIs occurred in the Surgical Wards (14 cases), followed by the ICU (10 cases).

The most frequent HAI types were *Clostridioides difficile* infections (9 cases, 33.3%) and catheter-associated urinary tract infections (8 cases, 29.6%). Other infections included ventilator-associated pneumonia, surgical site infections, and other respiratory infections (3 cases each, 11.1%), as well as bloodstream infections (1 case). The category “other respiratory infections” included SARS-CoV-2 respiratory cases.

Microbiological analysis identified multidrug-resistant organisms (MDROs), including carbapenem-resistant *Enterobacterales* (CRE) and methicillin-resistant *Staphylococcus aureus* (MRSA). A total of 9 HAIs were associated with MDROs, predominantly in the ICU and Surgical Wards (Table 6).

Among identified pathogens, Gram-negative microrganisms predominated (88.9%). *Klebsiella pneumoniae* was the most frequently isolated organism (44.4%), followed by *Klebsiella oxytoca* (22.2%), while *Escherichia coli*, *Pseudomonas aeruginosa*, and *Staphylococcus aureus* were each identified in 11.1% of cases (Table 7).

**Figure 4:**
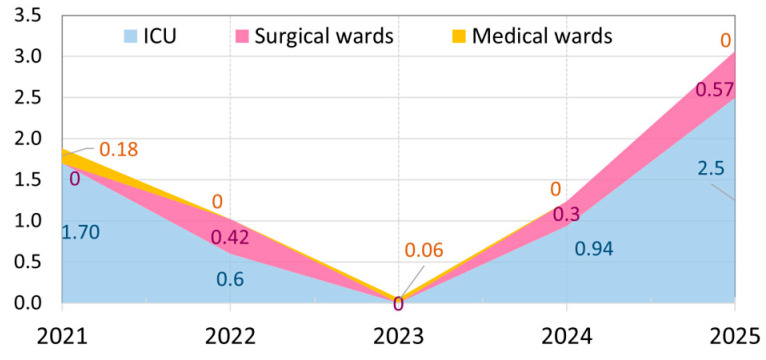
Annual healthcare-associated infection (HAI) incidence rates by hospital unit, 2021–2025.

The annual HAI rate remained low throughout the study period, ranging from 0.04 per 1000 patient-days in 2023 (1 case) to 0.46 per 1000 patient-days in 2025 (10 cases), with a five-year average of 0.23 per 1000 patient-days (Figure 4).

Nearly half of all HAIs were device- or procedure-associated infections (48.2%). A total of 13 such infections were identified, including ventilator-associated pneumonia (VAP), catheter-associated urinary tract infections (CAUTI), and surgical site infections (SSI). Urinary tract infections (UTI) had the highest rate per exposure (0.59%, *n* = 8), followed by VAP (0.11%, *n* = 3) and SSI (0.02%, *n* = 2) (Table A1).

Differences between Table 6 and Appendix Table A1 reflect distinct classification frameworks and denominator structures. Table 6 includes all wound-related infections recorded in the surveillance system, whereas Table A1 includes only ECDC-defined postoperative surgical site infections with available procedure-based denominators.

### 3.5. Exploratory Ecological Correlation Between Antibiotic Consumption and HAI Rates

An exploratory descriptive assessment of rank-based co-variation between antibiotic consumption and HAI rates was performed using Spearman’s rank correlation coefficient based on annual aggregated data (2021–2025).

Given the very limited number of annual observations (*n* = 5 per unit) and the low absolute number of HAI events (*n* = 27 over the entire study period), this analysis does not allow for statistical inference and should not be interpreted in terms of hypothesis testing or causal relationships. The results are therefore presented solely as descriptive measures of temporal co-variation.

Overall, no consistent or robust patterns of association were identified between total antibiotic consumption and HAI rates across hospital units.

At the unit level, numerical coefficients suggested an unstable positive association in the ICU (r = 0.17) and a numerically higher but unstable coefficient in the Surgical Wards (r = 0.60), while a negligible negative coefficient was observed in the Medical Wards (r = −0.04). At the hospital level, a weak positive coefficient was identified (r = 0.19) (Table 8). Given the very small sample size and high inter-annual variability, these coefficients should be interpreted strictly as descriptive measures of co-variation rather than inferential estimates.

Table 8 summarizes correlation coefficients between antibiotic consumption and HAI rates across hospital units. Table 9 provides a descriptive overview of mean antibiotic consumption and mean HAI rates across units over the study period. Marked heterogeneity was observed in antibiotic consumption between units, with the highest mean values in the Medical Wards and the lowest in the ICU, whereas HAI rates were highest in the ICU and substantially lower in the other wards.

These differences likely reflect variation in patient case-mix, severity of illness, and intensity of clinical activity across hospital units. However, given the ecological design and aggregated nature of the data, these descriptive patterns should not be interpreted as evidence of a direct relationship between antibiotic consumption and HAI incidence.

Consequently, these results should be interpreted strictly as contextual and exploratory observations within a surveillance dataset and not as analytical support for the correlation estimates presented in Table 8. No individual-level or causal inferences can be made from these data.

### 3.6. Exploratory Correlation Between Antibiotic Classes and Specific HAI Types

Exploratory descriptive rank-based co-variation metrics were used to assess ecological relationships between antibiotic consumption by class and specific healthcare-associated infection (HAI) types at unit level over the study period (2021–2025).

Given the very limited number of annual observations (*n* = 5 per unit), these coefficients are highly unstable, substantially underpowered, and highly sensitive to minor inter-annual fluctuations. Accordingly, all results should be interpreted strictly as hypothesis-generating outputs without statistical or inferential meaning.

Numerically elevated co-variation was observed between fluoroquinolone consumption and *Clostridioides difficile* infection (CDI) incidence (r = 0.89). However, due to the extremely limited number of time points, the ecological design, and the aggregated nature of the dataset, this coefficient should not be interpreted as an effect estimate or evidence of a causal relationship.

Additional exploratory numerical patterns were observed between penicillins combined with β-lactamase inhibitors and urinary tract infections (r = 0.54), as well as between polymyxin consumption and ICU-associated HAIs (r = 0.76) (Table 10). These coefficients were not consistent across sensitivity perspectives and should be interpreted strictly as descriptive indicators of temporal co-variation rather than analytical associations.

Overall, these analyses are constrained by the ecological study design, aggregated annual data structure, and extremely limited temporal resolution. Consequently, no causal or individual-level inferences can be drawn, and all reported coefficients should be regarded solely as unstable hypothesis-generating descriptive indicators.

### 3.7. Unit-Level Differences and Descriptive Surveillance Patterns Across the Study Period

Descriptive heterogeneity in antibiotic consumption and HAI incidence was observed across hospital units. The ICU showed higher relative use of broad-spectrum and Reserve antibiotics, including carbapenems, glycopeptides, oxazolidinones, and polymyxins. In contrast, fluoroquinolones, penicillins, and cephalosporins were more frequently used in the Medical and Surgical Wards (Table A2).

The distribution of HAI types also showed variability across units. *Clostridioides difficile* infections (CDI) and catheter-associated urinary tract infections (CAUTI) were among the most frequently reported infection types across the study period, with higher absolute numbers observed in the Surgical Wards and ICU (Table A3).

The distribution of HAI types also varied across hospital units. *Clostridioides difficile* infections (CDI) and catheter-associated urinary tract infections (CAUTI) were among the most frequently reported infection types during the study period, with higher absolute numbers observed in the Surgical Wards and ICU (Table A3). Device- and procedure-associated infections, including ventilator-associated pneumonia (VAP), CAUTI, and surgical site infections (SSI), were identified in the corresponding clinical settings.

Overall, these findings describe unit-level heterogeneity in antibiotic consumption patterns and reported HAI distribution over the study period. However, given the ecological design, aggregated annual data structure, and potential under-ascertainment of HAIs within routine surveillance systems, these descriptive patterns should not be interpreted as evidence of causal relationships or as indicators of the effectiveness of antimicrobial stewardship interventions.

### 3.8. Summary of Key Findings

- Descriptive heterogeneity in antibiotic consumption was observed across hospital units, with relatively higher use of broad-spectrum antibiotic classes in higher-risk clinical settings;
- Higher healthcare-associated infection (HAI) rates were recorded in the ICU compared with other hospital units; however, these estimates are based on small numbers and should be interpreted within the limitations of potential under-ascertainment, surveillance variability, and ecological aggregation;
- Exploratory ecological analyses identified numerically elevated temporal co-variation between fluoroquinolone consumption and *Clostridioides difficile* infection (CDI), while other correlations between antibiotic classes and specific HAI types were inconsistent and should be interpreted strictly as unstable hypothesis-generating descriptive observations.

## 4. Discussion

The present study provides an overview of antibiotic consumption patterns and healthcare-associated infection (HAI) surveillance data across multiple hospital units over a five-year period. The analysis describes variability in antimicrobial use between clinical units, with predominance of broad-spectrum antibiotics and heterogeneous HAI distribution, particularly in high-risk settings such as the Intensive Care Unit.

### 4.1. Antibiotic Consumption Patterns and Trends

The present study demonstrated interannual variability in antibiotic consumption between 2021 and 2025, with heterogeneous changes across antibiotic classes and hospital units. Overall, the observed patterns suggest a relatively stable underlying prescribing structure with short-term fluctuations, particularly in 2022.

Watch antibiotics accounted for a substantial proportion of total antibiotic use and exceeded Access antibiotics during the 2023–2025 period. In contrast, Reserve antibiotics represented a relatively small proportion of overall consumption, with higher proportions observed in high-risk areas such as the ICU and surgical wards. These patterns are consistent with European surveillance data from ECDC and ESAC-Net, which describe a persistent predominance of Watch-group antibiotics in acute-care hospitals across the EU/EEA (approximately 45–60% of hospital antibiotic consumption) [1,29,33].

Access antibiotics consumption remained below WHO-recommended AWaRe targets of ≥60%, indicating a distribution not fully aligned with WHO AWaRe stewardship recommendations [25,26].

The interannual variability, including the peak in 2022, is consistent with fluctuations in hospital antibiotic consumption reported in European surveillance systems, where changes in case mix, infection pressure, and healthcare activity have been associated with short-term variability in antimicrobial use [1,33]. In the present study, this peak likely reflects a combination of post-pandemic prescribing patterns, ongoing COVID-19-related hospital activity, and the early phase of antimicrobial stewardship implementation. Empirical antibiotic use was particularly common in patients with suspected or confirmed COVID-19 infection during this period, especially in medical wards with dedicated COVID-19 care areas.

The subsequent decrease in antibiotic consumption observed after 2022 may reflect a normalization of hospital activity together with progressive implementation of antimicrobial stewardship measures during the study period. Institutional prescribing recommendations based on local microbiological surveillance data were implemented at the end of 2022 and updated in 2024, alongside the expansion of audit-and-feedback activities and educational initiatives aligned with the WHO AWaRe classification. Similar associations between stewardship activities and changes in antibiotic consumption have been reported in the literature [20,21,25,29,34,35]. However, given the observational design of the study, no causal interpretation can be made.

The relatively high proportion of antibiotics with broad Gram-negative activity, particularly fluoroquinolones and extended-spectrum cephalosporins, is consistent with global and European surveillance reports [2,7,8,33]. These data highlight the continued predominance of Watch-group antibiotics in acute-care hospital settings.

Overall, the observed AWaRe distribution aligns with findings from multinational point prevalence surveys and ESAC-Net analyses describing persistent challenges in optimizing antibiotic selection in hospital settings [29,33,35]. The use of the Defined Daily Dose (DDD) methodology supports comparability with international surveillance data [27,28,39].

### 4.2. Unit-Level Variability in Antibiotic Use

Significant differences in antibiotic consumption were observed between hospital units. The Medical Wards accounted for the largest share of total antibiotic use, in agreement with European surveillance data indicating that general medical wards represent major contributors to inpatient antimicrobial consumption due to patient volume and frequency of infectious disease management [18,19,33].

The Intensive Care Unit (ICU), despite representing a smaller proportion of total antibiotic use, showed a higher relative consumption of broad-spectrum and Reserve antibiotics, including carbapenems, glycopeptides, oxazolidinones, and polymyxins. This pattern is consistent with ECDC ICU surveillance data and European point prevalence studies, describing intensive care units as settings with high antimicrobial consumption, associated with greater patient acuity, frequent use of invasive devices, and increased prevalence of multidrug-resistant organisms [1,10,11,38,40]. The relative proportion of broad-spectrum antibiotic use observed in the ICU suggests greater dependence on these agents compared with stewardship-oriented prescribing benchmarks reported in European surveillance frameworks, emphasizing the complexity of antimicrobial management in critical care settings.

Surgical Wards showed an intermediate antibiotic consumption profile, with predominant use of cephalosporins and penicillins, likely reflecting perioperative prophylaxis and postoperative infection management. Similar prescribing distributions have been described in European studies of surgical antibiotic use, where beta-lactam antibiotics remain the most frequently used classes [12,13,15,33].

These unit-specific differences are consistent with expected variation in clinical activity across hospital settings and provide relevant contextual information for antimicrobial stewardship planning. In particular, the higher relative use of broad-spectrum and Reserve antibiotics in the ICU identifies an important area for targeted stewardship efforts, in line with ECDC recommendations emphasizing ICU-oriented optimization strategies [38].

### 4.3. Healthcare-Associated Infections and MDROs

The overall healthcare-associated infection (HAI) rate observed in this study was lower than estimates reported in European acute-care hospital surveillance systems. According to ECDC point prevalence surveys (PPS), approximately 5–7% of hospitalized patients in EU/EEA acute-care hospitals are estimated to acquire at least one HAI, with higher prevalence typically reported in intensive care and surgical wards [1,33]. These PPS-derived estimates are not directly comparable with the incidence-based rates used in the present study, which are expressed per 1000 patient-days and derived from routine institutional surveillance.

The markedly lower observed incidence compared with European acute-care benchmarks likely reflects methodological differences inherent to routine surveillance systems, including variability in diagnostic intensity, case ascertainment, and surveillance sensitivity, as well as potential under-detection or under-reporting of cases. Therefore, the reported values should be interpreted as HAIs detected within the institutional surveillance framework rather than an estimate of the full underlying infection burden.

Consistent with international surveillance data, including ECDC reports, the ICU showed the highest HAI incidence, in line with the characteristics of critically ill patient populations and the frequent use of invasive devices [1,38]. However, this pattern should be interpreted with caution, as it may also be influenced by differences in patient severity, invasive device utilization, diagnostic intensity, surveillance practices, and denominator definitions across hospital units.

The distribution of infection types was consistent with European epidemiological patterns, with *Clostridioides difficile* infections and catheter-associated urinary tract infections representing the most frequently reported HAIs [9,31]. Nearly half of all infections were device- or procedure-associated, consistent with international data highlighting the central role of invasive procedures in HAI epidemiology [21,22].

To improve contextualization of device- and procedure-associated infection burden, additional analyses were performed using procedure- and device-based denominators (Table A1). However, these indicators are not methodologically comparable to standard incidence density metrics used in ECDC and NHSN surveillance systems, which are based on device-days. Consequently, direct comparison with international benchmarks should be avoided, and these measures should be interpreted solely as internal institutional surveillance indicators.

The detection of multidrug-resistant organisms (MDROs), including carbapenem-resistant *Enterobacterales* and methicillin-resistant *Staphylococcus aureus* (MRSA), is consistent with the global burden of antimicrobial resistance in healthcare settings [4,41]. Previous studies have described associations between antimicrobial exposure and the selection of multidrug-resistant Gram-negative organisms in hospital settings; however, such relationships cannot be assessed within the design of the present study [42,43].

MDRO classification was based exclusively on routine antimicrobial susceptibility testing performed in the hospital microbiology laboratory according to Clinical and Laboratory Standards Institute (CLSI) breakpoints. Only antimicrobial categories tested in routine practice were included, which may lead to underestimation of MDRO prevalence due to incomplete susceptibility panels. All MDROs were identified among clinically confirmed HAI episodes; no screening-based colonization cases were included.

Taken together, these findings should be interpreted within the context of important surveillance and case ascertainment limitations. Given the tertiary-care setting, the very low number of reported HAIs, and the reliance on routine surveillance systems, the dataset likely reflects infections detected through institutional reporting mechanisms rather than the true underlying burden of healthcare-associated infections. Variability in diagnostic intensity, reporting practices, and surveillance sensitivity across hospital units and over time may therefore have substantially influenced the magnitude and direction of observed incidence patterns, limiting causal interpretation.

Finally, due to the ecological design of the study and the use of aggregated annual data, no individual-level inferences can be made. Observed associations should therefore be interpreted as exploratory and hypothesis-generating, rather than causal or representative of true infection estimates.

### 4.4. Exploratory Relationship Between Antibiotic Use and HAIs

The analysis identified weak and inconsistent correlations between total antibiotic consumption and HAI rates at both unit and hospital levels. These findings are consistent with ecological evidence indicating that observed associations between antibiotic use and infection rates are influenced by multiple contextual factors, including case mix and surveillance sensitivity [5,6,44]. The limited number of observations and potential variability in case detection may have further influenced the observed patterns.

An isolated numerically high and unstable co-variation coefficient was observed between fluoroquinolone use and CDI incidence. While this pattern is consistent with previously reported associations in the literature, it should be interpreted cautiously given the ecological design, the small number of observations, and the absence of inferential statistical power in the present dataset.

Other observed associations did not show consistent patterns across units or time. Previous studies have similarly highlighted potential links between antibiotic consumption and healthcare-associated infections, particularly those caused by multidrug-resistant organisms, while emphasizing the limitations of causal inference in ecological analyses [42,44].

Overall, these findings indicate that total antibiotic consumption alone may not adequately reflect the complexity of HAI dynamics in acute-care hospital settings.

### 4.5. Implications for Antimicrobial Stewardship

The predominance of Watch antibiotics and the relatively low proportion of Access antibiotics suggest suboptimal alignment with WHO stewardship targets [25,26]. Increasing the use of Access antibiotics remains a key objective of antimicrobial stewardship programs. The observed changes in antibiotic use over time may be partly associated with the progressive implementation of stewardship activities during the study period, including formulary restriction policies for selected broad-spectrum antibiotics, prescription audits with feedback, educational activities on appropriate empirical therapy and the WHO AWaRe classification, and updates of local prescribing recommendations based on local resistance patterns. In this context, monitoring of AWaRe distribution may provide a useful indicator for stewardship-oriented quality improvement.

Similar trends have been reported in studies evaluating stewardship programs, which demonstrate improvements in prescribing practices and reductions in inappropriate antibiotic use [20,34,35]. In the present study, stewardship activities were implemented as part of routine institutional practice across hospital units, with greater intensity in higher-acuity settings such as the ICU and Surgical Wards. However, given the observational design and the absence of a structured interventional framework, the independent effect of specific stewardship measures on antibiotic consumption patterns or HAI rates cannot be determined.

The heterogeneity observed across hospital units highlights the need for tailored, unit-specific stewardship strategies. ICU settings, in particular, require intensified monitoring due to higher patient acuity and broader-spectrum antibiotic use. These findings are consistent with broader European efforts to strengthen antimicrobial resistance surveillance and optimize antibiotic use through coordinated policy frameworks and monitoring systems [45]. Furthermore, integrated approaches are required to address the complex interplay between antimicrobial use and resistance at both hospital and community levels [43].

Future research using quasi-experimental designs may help better evaluate the impact of targeted antimicrobial stewardship interventions on antibiotic consumption and infection outcomes.

### 4.6. Strengths and Limitations

This study has several strengths, including the multi-year observation period, the inclusion of multiple hospital units, and the integrated evaluation of antibiotic consumption and HAI surveillance data within a tertiary emergency hospital setting.

However, several limitations should be acknowledged. First, the observational design does not allow causal inferences. In addition, the relatively small number of HAI events may have limited the statistical power to detect moderate or weak associations between antibiotic consumption and infection rates.

The low HAI incidence observed during the study period likely reflects, at least in part, limitations inherent to routine surveillance systems, including potential under-detection or under-reporting, variability in surveillance intensity, and differences in diagnostic practices across hospital units. Although surveillance was conducted according to standardized ECDC definitions and institutional infection prevention and control (IPC) protocols, variability in routine case ascertainment may still have existed, particularly outside high-risk settings such as the ICU. Therefore, the analyzed dataset should be interpreted as reflecting detected and reported HAIs rather than the true underlying burden of healthcare-associated infections.

Important potential confounding factors were not fully captured in this analysis, including patient case mix, severity of illness, device utilization, and variability in IPC practices across hospital units. These factors may have influenced both HAI incidence and the observed associations between antibiotic consumption and infection rates. This limitation is particularly relevant when comparing ICU and non-ICU units, where differences in invasive device exposure and patient severity may substantially affect infection risk.

The use of aggregated routinely collected data further limited adjustment for individual-level risk factors and may have resulted in incomplete capture of less severe or clinically unrecognized infections. Therefore, the observed associations should be interpreted as exploratory and hypothesis-generating rather than causal.

Antibiotic consumption data were based on pharmacy dispensing records rather than direct patient-level administration data. Consequently, dispensed quantities may not fully correspond to actual antibiotic exposure due to ward stock management, medication returns, treatment modifications, or incomplete administration. Furthermore, although the Defined Daily Dose (DDD) methodology is widely used for antimicrobial surveillance and international benchmarking, it has recognized limitations in specific patient populations, particularly critically ill patients and those requiring dose adjustments. In such settings, DDD metrics may not accurately reflect administered doses or treatment intensity. Patient-level Days of Therapy (DOT) data were not consistently available across all hospital units during the study period and therefore could not be systematically analyzed.

Device- and procedure-associated infection rates were analyzed using procedure- and device-based denominators when available. However, standardized device-day surveillance data were not consistently available across all hospital units throughout the study period, precluding systematic calculation of incidence densities per 1000 device-days.

Overall, the findings of this study should be interpreted as surveillance-oriented and hypothesis-generating observations derived from routinely collected hospital data. Given the limitations described above, particularly potential under-ascertainment of HAIs and residual confounding, the observed associations between antibiotic consumption and HAI incidence should not be interpreted as evidence of causality or as definitive estimates of infection burden.

### 4.7. Future Directions

Future studies should include larger datasets and longer observation periods to better characterize temporal patterns and ecological associations. Integration of patient-level data and more detailed microbiological analyses would provide additional insight into the relationship between antibiotic use, healthcare-associated infections, and antimicrobial resistance.

Extension of the observation period and inclusion of additional data sources may further improve the robustness of temporal trend analyses and allow exploration of factors potentially associated with variability in antibiotic consumption and HAI indicators.

This study contributes real-world surveillance data from a multi-unit emergency hospital in Eastern Europe, a setting that remains underrepresented in antimicrobial stewardship and HAI surveillance literature.

## 5. Conclusions

This study provides a descriptive evaluation of antibiotic consumption patterns and healthcare-associated infection (HAI) surveillance in a multi-unit emergency hospital over a five-year period. The findings demonstrated a predominance of broad-spectrum antibiotics and a consistently low proportion of Access antibiotics according to the WHO AWaRe classification.

Marked heterogeneity in antibiotic consumption was observed across hospital units, with higher overall consumption in the Medical Wards and greater relative use of broad-spectrum and Reserve antibiotics in the ICU. Reported HAI incidence was low overall, with higher rates observed in critical care settings; however, these findings should be interpreted within the limitations of routine surveillance systems and the possibility of under-ascertainment.

Exploratory ecological descriptive analyses did not identify consistent or robust associations between total antibiotic consumption and HAI rates across hospital units. Numerically elevated temporal co-variation was observed between fluoroquinolone consumption and *Clostridioides difficile* infection (CDI); however, this finding should be interpreted strictly as exploratory and hypothesis-generating given the ecological design, aggregated annual data, and limited number of observations.

Overall, these findings support the value of continuous institutional surveillance of antibiotic use and HAIs while emphasizing the methodological limitations inherent to aggregated ecological analyses. Future surveillance systems integrating patient-level antimicrobial administration data, standardized HAI ascertainment methods, and longitudinal analytical approaches may improve the accuracy and interpretability of antimicrobial use and HAI surveillance in complex hospital settings.

Further studies using larger datasets, standardized surveillance methodologies, and more robust longitudinal designs are required to better characterize relationships between antimicrobial consumption and infection-related outcomes.

## Figures and Tables

**Table 1:** Characteristics of hospital units and surveillance systems included in the study.

Unit	Number of Beds	Average Occupancy Rate (%) ^1^	Patient Type	Surveillance Systems Used
ICU ^4^	15	33.3	Critical care	HAI surveillance; MDRO surveillance; Device- and procedure-associated infection surveillance
Emergency Department	5	n/a ^2^	Acute admissions	MDRO surveillance
Surgical Wards	88	34.1	Postoperative care	HAI surveillance; MDRO surveillance; Device- and procedure-associated infection surveillance
Medical Wards	87	43.1	Internal medicine, Dermatology, Psychiatry, Neurology	HAI surveillance; MDRO surveillance
Day-care Hospitalization Unit	10	100 ^3^	Short stays care	MDRO surveillance

^1^ Average occupancy rate calculated as mean over study period. ^2^ Not applicable due to high patient turnover. ^3^ Calculated based on daily turnover (full utilization). ^4^ Abbreviations: ICU—intensive care unit; HAI—healthcare-associated infections; MDRO—multidrug-resistant organism; n/a—not applicable.

**Table 2:** Antibiotic consumption trends (DDD per 100 bed-days), 2021–2025.

Class of Antibiotics	DDD Per 100 Bed-Days				
	2021	2022	2023	2024	2025 ^1^
Aminoglycosides	18.36	59.00	18.68	7.80	13.65
Antipseudomonal penicillins/β-lactamase inhibitors	13.54	32.15	11.36	7.13	8.24
Carbapenems	16.68	63.06	12.73	11.32	25.55
Extended-spectrum cephalosporins	35.09	174.38	39.05	26.24	52.95
Fluoroquinolones	46.69	273.97	54.80	62.67	123.29
Folate pathway inhibitors	14.00	0	0	0	0
Glycopeptides	0 ^2^	76.32	2.93	10.61	5.57
Nitroimidazoles	14.83	85.49	8.54	10.76	0
Lincosamides	9.75	17.01	7.86	6.84	12.42
Macrolides	31.43	128.50	14.80	16.15	15.00
Nitrofurans	0	0	0	7.00	20.83
Non-extended spectrum cephalosporins	0	169.36	13.53	7.05	9.14
Oxazolidinones	18.16	104.71	5.60	7.28	44.11
Penicillins	34.65	228.86	33.81	17.77	50.08
Penicillins/β-lactamase inhibitors	16.50	107.69	24.10	37.53	60.56
Polymyxins	2.02	7.08	0.57	0.74	0.25
Tetracyclines	22.81	77.53	17.42	23.27	22.67
Total	294.51	1605.11	265.78	260.16	464.31

^1^ Year 2025 represents a partial year (January–October) and is not directly comparable with full-year estimates. Values should be interpreted as provisional consumption estimates. ^2^ Zero values indicate no recorded consumption during the respective year. Abbreviations: DDD—defined daily dose.

**Table 3:** Exploratory linear trend summary (2021–2025).

Class of Antibiotics	β (Slope Per Year)	R^2^
Aminoglycosides	−6.062	0.224
Antipseudomonal penicillins/β-lactamase inhibitors	−3.562	0.334
Carbapenems	−3.400	0.059
Extended-spectrum cephalosporins	−11.242	0.078
Fluoroquinolones	−5.810	0.009
Folate pathway inhibitors	−2.800	0.500
Glycopeptides	−5.457	0.072
Nitroimidazoles	−10.439	0.224
Lincosamides	−0.483	0.035
Macrolides	−14.521	0.217
Nitrofurans	4.866	0.722
Non-extended spectrum cephalosporins	−14.403	0.098
Oxazolidinones	−4.553	0.030
Penicillins	−18.023	0.113
Penicillins/β-lactamase inhibitors	1.796	0.006
Polymyxins	−0.988	0.338
Tetracyclines	−5.454	0.119
Total	−100.535	0.081

Antibiotic consumption data are presented descriptively. Linear trend estimates were calculated using simple linear regression solely as an exploratory tool to summarize the direction and magnitude of change over time. Given the short and highly variable time series, results should not be interpreted as evidence of statistically robust or definitive temporal trends. Abbreviations: β—(beta)—slope estimate (change per year); R^2^—Coefficient of determination.

**Table 4:** Distribution of total antibiotic consumption by hospital unit.

Hospital Unit	Antibiotic Consumption (DDD Per 100 Bed-Days)	% of Total Consumption
Medical Wards	1779.64	61.58
Surgical Wards	649.65	22.48
Intensive Care Unit	349.36	12.08
Emergency Department	40.75	1.41
Day-care Hospitalization Unit	70.47	2.43
Total	2889.87	100

Antibiotic consumption values are expressed as Defined Daily Dose per 100 bed-days (DDD/100 bed-days). Percentages represent the proportional contribution of each hospital unit to total hospital antibiotic consumption.

**Table 5:** Antibiotic consumption intensity by hospital unit (DDD/100 bed-days).

Class of Antibiotics	ICU	Surgical Wards	Medical Wards	Emergency Department	Day-Care Unit
Aminoglycosides	23.50	36.87	54.18	0.33	2.61
Antipseudomonal penicillins/β-lactamase inhibitors	17.16	18.50	36.76	0.00	0.00
Carbapenems	34.21	39.19	55.94	0.00	0.00
Extended-spectrum cephalosporins	33.85	76.16	184.54	9.08	24.08
Folate pathway inhibitors	1.00	0.00	0.00	0.00	0.00
Fluoroquinolones	58.91	116.18	370.47	9.51	6.35
Glycopeptides	33.38	23.20	38.85	0.00	0.00
Nitroimidazoles	21.71	22.19	67.72	6.00	2.00
Lincosamides	9.88	21.17	22.83	0.00	0.00
Macrolides	0.00	40.09	165.79	0.00	0.00
Nitrofurans	0.00	0.00	27.82	0.00	0.00
Non-extended spectrum cephalosporins	14.48	26.93	147.67	9.00	1.00
Oxazolidinones	42.56	77.57	73.73	0.00	0.00
Penicillins	34.17	92.86	204.38	1.00	32.76
Penicillins/β-lactamase inhibitors	16.62	55.58	166.68	5.83	1.67
Polymyxins	2.10	1.16	7.40	0.00	0.00
Tetracyclines	6.83	2.00	154.87	0.00	0.00

Values are expressed as DDD/100 bed-days and reflect antibiotic consumption intensity within each hospital unit. Abbreviations: ICU—intensive care unit.

**Table 6:** Healthcare-Associated Infections and Incidence Rates by Hospital Unit.

Unit	Total HAI	% of Total HAI	HAI Rate ^1^ (Per 1000 Patient-Days)	CDI	UTI	BSI	VAP	SSI	Other Respiratory Infections	MDRO-Associated HAIs (*n*)
ICU	10	37.04	1.13	2	3	1	3	0	1	5
Surgical Wards	14	51.85	0.26	4	5	0	0	3	2	4
Medical Wards	3	11.11	0.05	3	0	0	0	0	0	0
Total	27	100	0.27	9	8	1	3	3	3	9

^1^ HAI rates are expressed per 1000 patient-days. These rates are based on routine surveillance data and may be affected by under-ascertainment, variability in diagnostic practices, and differences in case detection across units and over time; therefore, they should be interpreted as descriptive indicators rather than precise epidemiological estimates or comparable surveillance benchmarks. Abbreviations: ICU—intensive care unit; CDI—*Clostridioides difficile* infection; UTI—urinary tract infection; BSI—bloodstream infection; VAP—ventilator-associated pneumonia; SSI—surgical site infection; MDRO—multidrug-resistant organism; HAI—healthcare-associated infection.

**Table 7:** Distribution and resistance profile of MDRO-associated HAIs.

MDRO	Resistance Profile	Number of HAIs	% of MDRO-Associated HAIs
*Klebsiella pneumoniae*	CRE	4	44.4
*Klebsiella oxytoca*	CRE	2	22.3
*Escherichia coli*	CRE	1	11.1
*Pseudomonas aeruginosa*	Carbapenem-resistant	1	11.1
*Staphylococcus aureus*	MRSA	1	11.1
Total	-	9	100

Abbreviations: MDRO—Multidrug-Resistant Organism; CRE—Carbapenem-Resistant *Enterobacterales*; MRSA—Methicillin-Resistant *Staphylococcus aureus*; HAIs—Healthcare-Associated Infections.

**Table 8:** Exploratory descriptive Spearman coefficients between antibiotic consumption and HAI rates by hospital unit (2021–2025).

Unit	Spearman Coefficient (r) ^1^
Intensive Care Unit	0.17
Surgical Wards	0.60
Medical Wards	−0.04
Total	0.19

^1^ Coefficients are based on annual aggregated data (*n* = 5 observations per unit) and should be interpreted strictly as exploratory descriptive measures of co-variation. Given the very small sample size and ecological study design, these values are not suitable for statistical inference or causal interpretation.

**Table 9:** Ecological descriptive mean antibiotic consumption and healthcare-associated infection rates by hospital unit (2021–2025).

Unit	Mean Antibiotic Consumption (DDD/100 Bed-Days) ^1^	Mean HAI Rate (Per 1000 Patient-Days)
Intensive Care Unit	69.51	1.15
Surgical Wards	93.88	0.26
Medical Wards	251.58	0.05
Total ^2^	160.04	0.22

^1^ Mean antibiotic consumption (DDD/100 bed-days) was calculated as a weighted mean across the study period (2021–2025). Mean HAI rates are based on routine surveillance data and should be interpreted as descriptive ecological indicators. ^2^ Total Hospital values are weighted means based on unit patient-days. Abbreviations: DDD—defined daily dose; HAI—healthcare-associated infection.

**Table 10:** Exploratory correlation between antibiotic classes and HAI types (Spearman correlation, 2021–2025).

Antibiotic Class	HAI Type	Spearman Coefficient (r) ^1^
Fluoroquinolones	CDI	0.89
Penicillins/β-lactamase inhibitors	UTI	0.54
Polymyxins	ICU-associated HAIs	0.76

^1^ Coefficients were calculated using annual aggregated data (*n* = 5 observations per series). These values are exploratory and ecological descriptive measures of co-variation and are not suitable for statistical inference or causal interpretation. Abbreviations: CDI—*Clostridioides difficile* infection; UTI—urinary tract infection; ICU—intensive care unit; HAI—healthcare-associated infection.

## Data Availability

Reasonable requests for data sharing should be addressed to the corresponding author.

## Abbreviations

The following abbreviations are used in this manuscript:

AMR	Antimicrobial Resistance
AMS	Antimicrobial Stewardship
API	Analytical Profile Index
AST	Antimicrobial Susceptibility Testing
ATC	Anatomical Therapeutic Chemical (classification system)
AWaRe	Access, Watch, Reserve (WHO antibiotic classification)
BSI	Bloodstream Infection
CAGR	Compound Annual Growth Rate
CDI	*Clostridioides difficile* Infection
CLSI	Clinical and Laboratory Standards Institute
CRE	Carbapenem-Resistant *Enterobacterales*
DDD	Defined Daily Dose
ECDC	European Centre for Disease Prevention and Control
ESBL	Extended-Spectrum Beta-Lactamase
HAI	Healthcare-Associated Infection
ICU	Intensive Care Unit
IPC	Infection Prevention and Control
MDROs	Multidrug-Resistant Organisms
MRSA	Methicillin-Resistant *Staphylococcus aureus*
NHSN	National Healthcare Safety Network
PPS	Point Prevalence Survey
SSI	Surgical Site Infection
UTI	Urinary Tract Infection
STROBE	Strengthening the Reporting of Observational Studies in Epidemiology
VAP	Ventilator-Associated Pneumonia
WHO	World Health Organization
β (beta)	Regression coefficient
R^2^	Coefficient of determination
r	Correlation coefficient
*p*	*p*-value

## Appendix A

### Appendix A.1

**Table A1:** Device- and procedure-associated healthcare-associated infections (HAIs) per 100 invasive procedures/exposures.

Type of Device- or Procedure-Associated HAI	Rate Per 100 Invasive Procedures/Exposures ^1^	Number of HAIs	Number of Invasive Procedures/Exposures
CAUTI	0.59	8	1354
VAP	0.11	3	2709
SSI	0.02	2	8121
Total	-	13	12,184

^1^ Rates were calculated as: (number of device- or procedure-associated HAI cases/number of corresponding invasive procedures or exposures) × 100. These indicators were calculated using procedure- and device-based denominators as proxies, as device-day data were not available in the surveillance database during the study period. Therefore, they are not directly comparable with ECDC/NHSN incidence density rates expressed per 1000 device-days. Abbreviations: CAUTI—catheter-associated urinary tract infection; VAP—ventilator associated pneumonia; SSI—surgical site infection; HAI—healthcare-associated infection.

### Appendix A.2

**Table A2:** Predominant antibiotic consumption classes by hospital unit and corresponding HAI indicators.

Unit	Top 3 Most Frequently Consumed Antibiotic Classes ^1^	Descriptive HAI Rate ^2^ (Per 1000 Patient-Days)	Number of HAI Cases
ICU	Carbapenems, Fluoroquinolones, Polymyxins.	1.13	10
Surgical Ward	Fluoroquinolones, Penicillins, Cephalosporins.	0.26	14
Medical Ward	Fluoroquinolones, Penicillins, Cephalosporins.	0.05	3
Emergency Department/Day-care Unit	Cephalosporins, Penicillins, Others.	n/a	0

^1^ Antibiotic consumption was expressed as Defined Daily Doses per 100 bed-days (DDD/100 bed-days) according to WHO ATC/DDD methodology. Top antibiotic classes represent the three most frequently consumed classes per unit over the study period. ^2^ HAI rates are presented descriptively per 1000 patient-days and should be interpreted within the limitations of the ecological study design and potential under-ascertainment. Abbreviations: ICU—intensive care unit; HAI—healthcare-associated infection; n/a—not applicable.

### Appendix A.3

**Table A3:** Antibiotic consumption classes by type of healthcare-associated infection (HAI).

HAI Type	Most Frequently Associated Antibiotic Classes ^1^	HAI Rate (Per 1000 Patient-Days)	Number of HAI Cases
CDI	Fluoroquinolones, Cephalosporins.	0.08	9
Urinary Tract Infection	Fluoroquinolones, Aminoglycosides.	0.07	8
Ventilator Associated Pneumonia	Carbapenems, Polymyxins.	0.03	3
Surgical Site Infection	Cephalosporins, Penicillins/β-lactamase inhibitors.	0.03	3

^1^ Antibiotic consumption was expressed as Defined Daily Dose per 100 bed-days (DDD/100 bed-days) according to WHO ATC/DDD methodology. Associations are presented descriptively and should not be interpreted as causal or inferential relationships. Abbreviations: CDI—*Clostridioides difficile* infection; HAI—healthcare-associated infection.

### Appendix A.4

**Table A4:** Antibiotic classification by therapeutic class (ATC J01) used for consumption analysis.

Therapeutic Class	Antibiotics Included
Penicillins	Benzylpenicillin, ampicillin, amoxicillin
Penicillins/β-lactamase inhibitors	Amoxicillin/clavulanate, ampicillin/sulbactam
Antipseudomonal penicillins/β-lactamase inhibitors	Piperacillin/tazobactam
Non–extended-spectrum cephalosporins	Cefalexin, cefazolin, cefuroxime
Extended-spectrum cephalosporins	Ceftriaxone, cefotaxime, ceftazidime
Carbapenems	Imipenem, meropenem
Fluoroquinolones	Norfloxacin, ciprofloxacin, levofloxacin, moxifloxacin
Aminoglycosides	Gentamicin, amikacin
Macrolides	Clarithromycin
Lincosamides	Clindamycin
Glycopeptides	Vancomycin
Polymyxins	Colistin
Oxazolidinones	Linezolid
Tetracyclines	Tetracycline, doxycycline
Folate pathway inhibitors	Trimethoprim-sulfamethoxazole
Nitroimidazoles	Metronidazole
Nitrofurans	Nitrofurantoin

Antibiotics were grouped exclusively for consumption analysis (DDD-based surveillance). This classification was not used for antimicrobial susceptibility interpretation or multidrug-resistant organism (MDRO) classification. Intrinsic resistance patterns and organism-specific testing limitations were not considered in this grouping.
